# Prediction of a deletion copy number variant by a dense SNP panel

**DOI:** 10.1186/1297-9686-44-7

**Published:** 2012-03-23

**Authors:** Naveen K Kadri, Patrick D Koks, Theo H E Meuwissen

**Affiliations:** 1 Department of Animal and Aquacultural Sciences, Norwegian University of Life Sciences, PO Box 5003, 1432 Ås, Norway; 2 Animal Breeding and Genomics Centre, Wageningen University, PO Box 338, 6700 AH Wageningen, the Netherlands

## Abstract

**Background:**

A newly recognized type of genetic variation, Copy Number Variation (CNV), is detected in mammalian genomes, e.g. the cattle genome. This form of variation can potentially cause phenotypic variation. Our objective was to determine whether dense SNP (single nucleotide polymorphisms) panels can capture the genetic variation due to a simple bi-allelic CNV, with the prospect of including the effect of such structural variations into genomic predictions.

**Methods:**

A deletion type CNV on bovine chromosome 6 was predicted from its neighboring SNP with a multiple regression model. Our dataset consisted of CNV genotypes of 1,682 cows, along with 100 surrounding SNP genotypes. A prediction model was fitted considering 10 to 100 surrounding SNP and the accuracy obtained directly from the model was confirmed by cross-validation.

**Results and conclusions:**

The accuracy of prediction increased with an increasing number of SNP in the model and the predicted accuracies were similar to those obtained by cross-validation. A substantial increase in accuracy was observed when the number of SNP increased from 10 to 50 but thereafter the increase was smaller, reaching the highest accuracy (0.94) with 100 surrounding SNP. Thus, we conclude that the genotype of a deletion type CNV and its putative QTL effect can be predicted with a maximum accuracy of 0.94 from surrounding SNP. This high prediction accuracy suggests that genetic variation due to simple deletion CNV is well captured by dense SNP panels. Since genomic selection relies on the availability of a dense marker panel with markers in close linkage disequilibrium to the QTL in order to predict their genetic values, we also discuss opportunities for genomic selection to predict the effects of CNV by dense SNP panels, when CNV cause variation in quantitative traits.

## Background

A recently recognized source of genomic structural variation called Copy Number Variation (CNV), is gaining interest in genomic studies. It is defined as a DNA segment that is 1 or more kb long and is present at a variable copy number in comparison with a reference genome [1]. CNV are shown to be functionally active in humans. They are responsible for phenotypic changes by altering gene dosage, disturbing coding sequences and perturbing long-range gene regulation [2]. With the discovery of CNV in the cattle genome [3-5] and their potential to cause variation in economically important traits, capturing the effects of CNV and other complex genotypes on phenotype becomes an important factor in the prediction of genetic values.

The aim of this study was to investigate whether a simple deletion CNV can be predicted from dense SNP genotyping data using a multiple regression approach, which, if successful, implies that genetic variation due to this deletion CNV can be predicted in an automated manner by dense SNP genotyping. To this end, we report the linkage disequilibrium (LD) of a bi-allelic deletion type of CNV (the locus varying in copy number, 2 = normal and 1 = deletion) with surrounding SNP to determine whether SNP can predict this simple CNV. A model to predict CNV from surrounding SNP is developed and its accuracy is tested by cross-validation. Prediction of CNV with high accuracy would eliminate the need for explicit detection and genotyping of simple CNV. Our approach is general and can be extended to more complex CNV, but estimation of the prediction accuracy of more complex CNV is outside the scope of this paper.

## Methods

### Genotypic data on SNP and CNV

The SNP and CNV genotypes for dairy cattle were provided by the Milk Genomics Project conducted at Wageningen University, The Netherlands. In the project, 2,000 Holstein Friesian cows (belonging to five large sire families with about 200 daughters and 50 small sire families with about 20 daughters) were genotyped for 50 000 SNP on the Illumina Infinium platform [6], using a custom array described by Charlier et al. [7]. The 2,844 SNP genotypes on bovine chromosome 6 with a median interval of 18 kb were used for CNV detection. Two algorithms, PennCNV (2008 Nov19 version) [8] and cnvPartition (v1.2.0, a plug in of Bead studio version 3; Illumina Inc.) [9] were used with default settings for the detection of CNV. In total, 476 samples showed CNV regions with PennCNV and 245 samples with cnvPartition.

**Figure 1:**
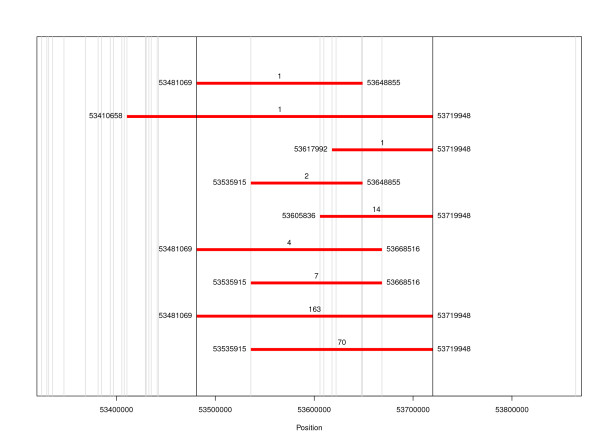
**Deletion variants detected in the 53 Mb region according to PennCNV**. The red bars show the SNP boundaries of the deletion with their position (bp); the number of animals detected with the variants is given at the top of the red bars and the vertical lines indicate the position of the SNP relative to the map of BTAU4 [11].

A bi-allelic deletion type CNV was detected on bovine chromosome 6 around 53 megabases (Mb) by both algorithms and was validated by significant evidence for Mendelian inheritance in 17 sire families. This common deletion CNV locus (a CNV region, CNVR [10]) was found to vary in copy number; two copies (normal) and one copy (deletion) and spanned 233 kb in the 53 Mb region. The CNV detection algorithms showed ambiguity in mapping the breakpoints of this CNV; the same deletions were detected with different boundaries both within and across families (Figure 1). We used the results from PennCNV for our study since it showed better compliance with the test of Mendelian inheritance. The boundaries of the variant detected in the majority of the animals (163) were considered to be the true boundaries of the CNV (53,481,069-53,719,948 bp in terms of map positions on bovine chromosome 6 on BTAU4 [11]). This CNVR was considered a validated deletion CNV to test our hypothesis.

Samples with missing genotypes were excluded from the analysis. This resulted in a dataset of CNV and SNP genotypes for 1,682 individuals, of which 263 carried the deletion. The dataset can be accessed in Additional file 1.

### Data analysis

**Figure 2:**
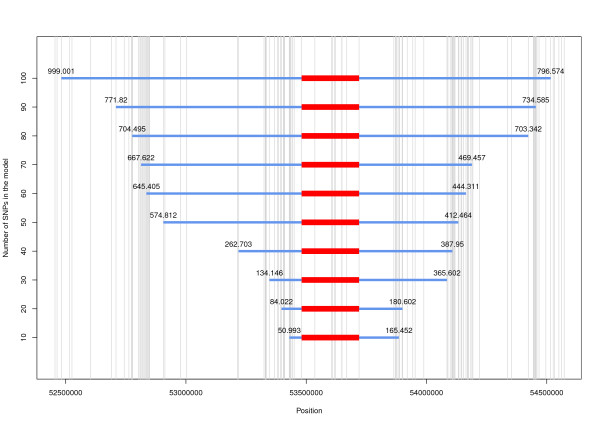
**Density of SNP (vertical lines) around the CNV region**. The red bar is the CNV region (53,481,069-53,719,948 bp) and the blue bar represents the region encompassing the SNP in different models; the distance of the farthest SNP from the CNV boundary (the region covered by the SNP) is given in kb above the blue bar.

Multiple linear regression analysis was carried out on the CNV using adjacent flanking SNP markers. Model (1) was run with different numbers of surrounding SNP markers (m = 10, 20, 30....100; with an equal number of markers to the left and right of the CNV):

(1) $$y=\mu 1_{n}+Xb+e$$

where **y **= n × 1 response vector of CNV genotypes, for n = 1,682 animals, coded as 1 for deletion and 2 for normal copy number; μ = overall mean; **1_n _**= vector of n ones; **X **= n × m matrix, with genotypes of n = 1,682 animals for m SNP markers; **b **= m × 1 vector of SNP effects on copy number; and **e **= vector of random residuals. The SNP carrying the "A" allele were encoded as the number of "A" alleles (AA = 2, AT/AC/AG = 1 and TT/CC/GG = 0). For one CG SNP, the number of "C" alleles was used for coding (CC = 2, CG = 1 and GG = 0). Model (1) was fitted using the SAS^® ^software [12]. The SNP were selected so that half of them were upstream and half were downstream from the CNV, starting adjacent to the CNV with 10 SNP and then further away up to 50 SNP on each side. Distances of SNP from the CNV borders are shown in Figure 2. The fraction of variation in copy number explained by the model was quantified by the coefficient of determination (R^2^) and the model accuracy (AM) was calculated as the square root of R^2^.

### Cross-validation

Ten-fold cross-validation was carried out to test the predictive accuracy of the model [13]. The data was randomly split into ten non-overlapping sample subsets. The data from nine subsets were used to fit the model with 10, 20, 30....100 SNP. The estimated SNP effects were then used to predict the copy number in the remaining 10th sub-set, which was excluded from the model fitting. This procedure was repeated for each of the 10 subsets, so that a prediction for every record was obtained once whilst it was excluded from the estimation model. The correlation between the predicted and observed copy number was calculated for each sample as a measure of accuracy (accuracy estimated by cross-validation; ACV) and was used to obtain the prediction accuracies with different numbers of SNP fitted in the model.

### Linkage disequilibrium

The linkage disequilibrium (LD) plot for the SNP and CNV was generated using HaploView (http://www.broadinstitute.org/haploview/haploview) [14]. The CNV was encoded as an SNP, as described elsewhere [15]; AT for deletion and TT for no deletion in the input file. The LD plot was also used to identify a "disconnected SNP" (dSNP, S173; see next section) that fell outside the tightly linked haplotype blocks, as defined by HaploView.

### Prediction of dSNP

To compare the prediction accuracy of the CNV by surrounding SNP with that of a certain single SNP, an SNP was predicted using the same model. Since the majority of the SNP were in tight LD blocks with r^2 ^~ 1, SNP genotypes for the dSNP S173 were included in the "**y**" vector and models (1) were fitted in SAS^®^. The R^2 ^of the dSNP prediction models were compared with those of the CNV prediction models.

## Results

### Linkage disequilibrium plot

**Figure 3:**
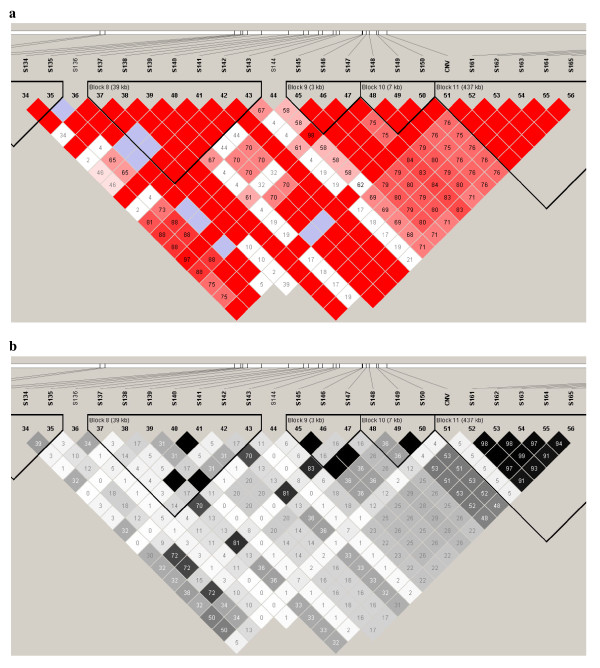
**a. Linkage disequilibrium plot (from HaploView) based on D' for the SNP surrounding the CNV**. Key: when LOD < 2, D' < 1 is white and D' = 1 is blue; when LOD > 2, D' < 1 is given in shades of pink/red and D' = 1 is given in bright red; the pair-wise D' values are given in the boxes. **b.** Linkage disequilibrium plot (from HaploView) based on r^2 ^for the SNP surrounding the CNV. Key: r2 = 0 is given in white, 0 < r2 < 1 is given in shades of grey and r2 = 1 is given in black.

HaploView was used to assess the LD pattern (Figure 3a and 3b) of the CNV with nearby SNP. Many SNP adjacent to the CNV region showed a D' of 1. However, no single SNP appeared to tag the CNV perfectly (r^2 ^= 1). Among the SNP with a D' value of 1, S135 (115 kb downstream the CNV boundary) showed the highest r^2 ^value of 0.13. The majority of the SNP showed tight LD with adjacent SNP (r^2 ^= D' = 1) (red triangles in Figure 3a).

### Prediction of the CNV

**Figure 4:**
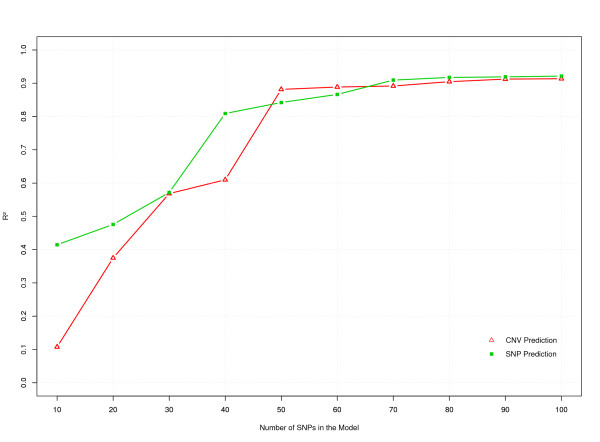
**R^2 ^of CNV (red triangles) and dSNP (green boxes) prediction models with different numbers of SNP**.

The CNV and SNP data on 1,682 animals were analyzed using model (1). This model used 10 to 100 SNP flanking the CNV. The fraction of the variance explained by the model (R^2^) increased with the number of SNP in the model (Figure 4). A low R^2 ^of 0.107 was obtained for the model with ten SNP but this increased gradually to 0.609 for a model with 40 SNP. A large increase in R^2 ^(0.27) was observed when the number of included SNP increased from 40 to 50. With 50 SNP, the model explained 88.1% of the variation in copy number.

Including even more SNP resulted in little increase in the value of R^2^, which reached a maximum value of 0.914 with 100 SNP. Since the curve was very flat between 50 and 100 SNP, we expect limited further increases in R^2 ^by extending the SNP panel beyond 100 SNP.

### Cross-validation

**Figure 5:**
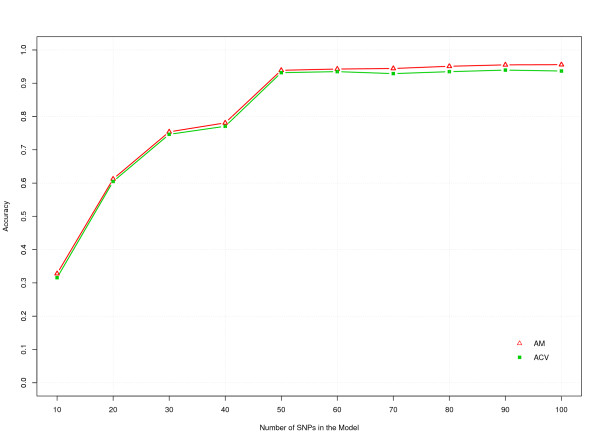
**The accuracies of predicting the CNV by surrounding SNP estimated by cross-validation (ACV, green boxes) and the model accuracies (AM, red triangles)**.

Cross-validation was carried out to assess the performance of the model by predicting CNV genotypes for samples that were excluded when fitting the model. The prediction accuracy estimated by cross-validation (ACV) was plotted against the model accuracy (AM), i.e. the accuracy of the predicted results by the model in Figure 5. The ACV closely followed the accuracy predicted by the model, AM, for different numbers of SNP and increased with an increasing number of SNP but was slightly lower compared to the accuracy predicted by the model. It reached a value of 0.93 with a 50 SNP model. Increasing the number of SNP in the model further, showed very little increase in ACV, which reached a maximum value of 0.94 for 100 SNP. Figure 5 also shows that the small increase in prediction accuracy, when increasing from 50 to 100 SNP, was even smaller for ACV than for AM.

### Prediction of dSNP

**Figure 6:**
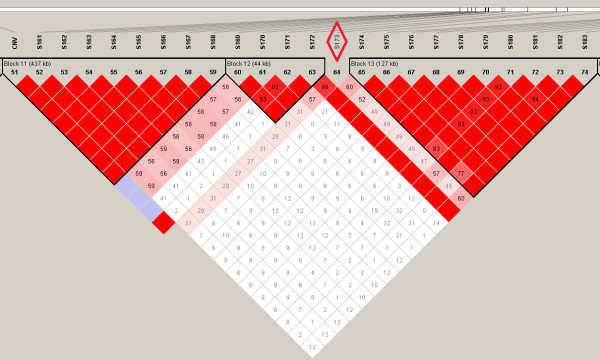
**Linkage disequilibrium plot based on D' for the SNP in the CNV region**. Key: when LOD < 2, D' < 1 is white and D' = 1 is blue; when LOD > 2, D' < 1 is given in shades of pink/red and D' = 1 is given in bright red; the pair-wise D' values are given in the boxes.

A disconnected SNP (dSNP, S173) that fell just between two tightly linked haplotype blocks (Figure 6) was compared to the CNV for its predictability. The SNP was predicted from 10 to 100 flanking SNP. The R^2 ^for the dSNP prediction model with different numbers of SNP is compared to that of the CNV in Figure 4. The models with 40 or less markers performed better for dSNP prediction than for CNV prediction. With 40 markers in the model, an R^2 ^of 0.81 was obtained for dSNP prediction compared to that of 0.61 for the CNV prediction model. However, with 50 SNP, a higher R^2 ^was obtained for the CNV prediction model (0.88) than for the dSNP prediction model (0.84). Thereafter, the R^2 ^for the dSNP prediction model closely followed the CNV prediction model, reaching a value of 0.92 for 100 SNP within 1,000 kb on each side of the CNV (Figure 2).

## Discussion

In this study, we have investigated the prediction of a CNV from surrounding SNP typed on a custom Illumina Infinium 50 k BeadChip, using a multiple regression model. Although the investigated CNV was discovered using specific CNV detection algorithms, we used it as a model for other, currently unknown CNV. We have assessed the accuracy with which an unknown CNV genotype can be predicted by predicting a common deletion CNV genotype using the surrounding SNP. The investigated CNV was a rather common large deletion CNV of 233 kb. We did not find any SNP in perfect LD (r^2 ^= 1), contrary to previous studies that reported strong LD for deletions with nearby SNP [15,16]. However, it was possible to predict the CNV with a high accuracy (0.94) by combining information from 50 or more flanking SNP in a multiple regression model. This accuracy was confirmed by cross-validation. If this result proves to be general, it can be concluded that the presence of a bi-allelic deletion type CNV and, in case it has causative effects, its related phenotypic effects, may be estimated by dense SNP genotyping with a high accuracy.

The in silico CNV detection algorithms used in the present study showed ambiguity in mapping the breakpoints (Figure 1). Seventy-seven of the CNV (out of 263 samples harboring a CNV in the 53 Mb region) started at position 53,535,915 on chromosome 6 (~54 kb downstream relative to the most common variant) and 14 CNV started at position 53,605,836, almost ~124 kb downstream (Figure 1). Thus, it is possible that there are multiple distinct CNV in this region. Similarly, there is an alternative CNV endpoint 5 kb upstream of the common CNV endpoint. This uncertainty in breakpoints might explain why we failed to find a SNP in perfect LD with the deletion region, although the CNV genotype calls seemed accurate since they showed Mendelian inheritance. Confirming the nature of the deletion and fine-mapping the CNV boundaries may help to detect better tag SNP for this region.

Perhaps a more likely reason for the relatively low LD between the CNVR and its surrounding SNP is the relatively large distance between the CNVR and the closest SNP, which may be general for CNV due to the often low SNP coverage in CNV regions (as shown in Figure 2). The first SNP downstream from the CNV was 39 kb away, whereas upstream, the first SNP was 145 kb away, which is far greater than the median SNP to SNP distance of 18 kb. Studies [15,16] that report SNP in strong LD with CNV use a denser SNP map and obtain perfect LD for nearby SNP. Thus, with the next generation SNP chips (containing ~700 k SNP), we expect to predict the CNV more accurately with fewer SNP. However, it is difficult to reliably detect SNP in CNV regions because of the genomic complexity that is generally found in the deleted or duplicated regions and the resulting low reliability of the reference sequence.

The accuracy of the model, as estimated by cross-validation, was high. The cross-validation accuracies were only slightly lower than those predicted by the statistical model. Thus, given a sufficiently big training data set, the model proved to be reliable for future predictions of deletion type CNV from SNP data.

A small increase in accuracy was observed for CNV prediction, when increasing from 50 to 100 SNP (Figure 5). The increase was much smaller for cross-validation accuracy than predicted by the model. Thus, the increase in R^2 ^(and AM) when increasing from 50 to 100 SNP is to a large extent due to over-fitting of the data by the model, and hardly results in a real increase in R^2 ^beyond 50 SNP. This suggests that the LD might be decreasing at distances >500 kb since the 50 SNP used in the model were within 500 kb from the CNV (Figure 2). This is consistent with a previous study that reported LD in eight breeds of cattle [17] and showed that the LD between pair-wise loci drops to background LD level at a distance of 500 kb.

We compared the predictability of the CNV with that of a 'disconnected' SNP (dSNP), S173. When using information from 40 or less flanking SNP, the SNP was predicted more accurately than the CNV. When including more SNP, the two models showed a similar R^2 ^pattern. Hence, it may be concluded that the predictability of a simple bi-allelic CNV follows the predictability of a dSNP when information from many (>50) SNP is used. This and the fact that the accuracies of the predictions of the CNV and S173 are almost identical with >50 SNP, suggest that both the CNV and dSNP may be on an extended haplotype that is predicted by the SNP with an accuracy of 0.94.

In this study, we have shown that a simple deletion CNV can be predicted with a high accuracy from neighboring SNP using a multiple regression approach. This suggests that dense SNP panels can capture the effects of this type of CNV. However, our study was limited to one large common deletion type CNV that was detected using CNV detection algorithms from SNP data, and a 50 K SNP chip that was solely targeted at SNP genotyping and generally has a poor coverage of CNV regions (as shown in Figure 2). Thus, although our approach is general, further studies are needed to investigate whether similar accuracies can be attained for other, more complex types of CNV.

Genomic selection relies on dense markers that jointly are in sufficiently high LD with (unknown) QTL, so that the effect of the QTL is accurately predicted by the sum of the SNP effects. This situation resembles very much our prediction of the CNV, in cases where the CNV causes quantitative trait genetic variation, and its position is not known. With a CNV of unknown position, we could not have selected the 100 nearest SNP, and we would have had to rely on all ~50 000 genome-wide markers to predict the CNV. Thus, in this case, the number of SNP effects would greatly exceed the number of records, which is known as the k>>n problem in statistics. Genomic selection deals with this problem by using informative prior distributions for the SNP effects. The accuracy of 0.94 found here is thus an upper bound for the accuracy of prediction of breeding values for a quantitative trait by the genomic selection approach, when the quantitative trait that is affected by the current CNV, possibly along with other CNV can be predicted with similar accuracy, and environmental effects. The prediction accuracy of 0.94 is an upper bound, because the k>>n problem may not be completely resolved by the prior distribution of SNP effects and the environmental effects reduce the accuracy of the estimates of the SNP effects relative to those in our study. Both these problems can be overcome by increasing the number of records, in which case the accuracy of genomic selection will approach this upper bound. A similar maximum accuracy of genomic selection was suggested by the result of Daetwyler [18]. With recent studies providing further evidence that CNV are associated with complex diseases in humans, designing genotyping chips with CNV probes may be important to increase the accuracy from the current ~90% towards 100% and thus to capture all genetic variation.

## Competing interests

The authors declare that they have no competing interests.

## Authors' contributions

NKK carried out the CNV detection, prediction study and drafted the manuscript. PDK designed and coordinated the CNV detection study. THEM conceived the CNV prediction study. All authors contributed in writing the manuscript, read and approved the final manuscript.

## Supplementary Material

Additional file 1 **Genotypic data on SNP and CNV**. The SNPs and CNV genotypes are given in the map order. CNV genotypes (with the header CNV) are coded as AT for deletion and AA for no deletion. Click here for file
